# Extracellular matrix composition modulates angiosarcoma cell attachment and proliferation

**DOI:** 10.18632/oncoscience.383

**Published:** 2017-12-07

**Authors:** Noel L. Shaheen, Esha Kataria, Jocelyn Antony, Dana Galvan, Yessenia Ballou, Brad A. Bryan

**Affiliations:** ^1^ Paul L. Foster School of Medicine, Texas Tech University Health Sciences Center, El Paso, Texas, USA; ^2^ Department of Biomedical Sciences, Texas Tech University Health Sciences Center, El Paso, Texas, USA

**Keywords:** angiosarcoma, extracellular matrix, fibronectin, collagen, endothelial

## Abstract

Angiosarcoma is a rare and generally fatal tumor composed of aberrant cells of endothelial origin. Because of its infrequency in humans, very little is known about the growth requirements of this vascular sarcoma. Unlike the rapidly proliferating solid tumors from which they are isolated from, many of the established angiosarcoma cell lines exhibit less than robust growth in culture and often fail to form tumors in xenograft models. In order to better understand angiosarcoma in vitro growth conditions, we focused on a singular aspect of their culture—adhesion to the extracellular matrix—in order to identify attachment substrates that may facilitate and/or enhance their growth in tissue culture. Our data indicates that the extracellular matrix of angiosarcomas contains similar protein compositions to that of non-diseased endothelial cells. Moreover, angiosarcoma cell lines exhibited strong attachment preference to substrates such as collagen I or fibronectin, and less preference to collagen IV, laminin, or tropoelastin. Growth on preferred extracellular matrix substrates promoted mitogenic signaling and increased proliferation of angiosarcoma cell lines. These findings provide insight that may lead to more successful in vitro growth of angiosarcoma cell lines.

## INTRODUCTION

Angiosarcoma is a rare and clinically highly variable malignant neoplasm composed of rapidly dividing, aggressively infiltrating cells of endothelial origin. The mortality rate of patients with angiosarcoma is exceptionally high as this tumor type responds very poorly to traditional treatment therapies, with a median survival time of between 3 to 8 months depending on TNM staging and location of the tumor [1, 2]. A wide variety of systemic therapies have been utilized to treat angiosarcomas, however the overall response rate is as low as 19% for traditional therapy [3-6] and ∼13% for novel anti-angiogenic treatments such as bevacizumab or sorafenib [7, 8], with a duration of response on the order of a few months for most treatments.

Due to the scarcity of angiosarcomas in humans, there are a very limited number of cell lines available to study [9-12] and many researchers studying these tumors rely heavily on engineered vascular tumor cell lines [13, 14] or canine hemangiosarcomas which are relatively common in certain species of dogs [15]. Purification of angiosarcoma cells from solid tumors remains a challenge, and once isolated many of these cells lines exhibit unexpectedly slow proliferation rates that are not at all reflective of their rapid and lethal dissemination in patients with the disease, and generally fail to recapitulate aggressive solid tumors in xenograft models. Methodologies that could facilitate the isolation and maintenance of angiosarcoma cell lines could greatly improve opportunities to discover effective treatments against this rare disease.

The extracellular matrix (ECM) is a highly variable protein-rich composition with unique physical properties that governs the fate of cells through biochemical and biomechanical processes via cell-to-matrix interactions. Components of the ECM are often used to coat glass or plastic surfaces to enhance *in vitro* cell attachment, and the nature of these ECM components plays an essential role in cell adhesion, migration, proliferation, and overall behavior. ECM surface coatings such as fibronectin or collagen are commonly used as cell culture substrates for endothelial cells and their progenitors, as primary endothelial cells generally fail to thrive on cell culture plastic alone [16]. Given the scarcity of data on optimum culture conditions for angiosarcoma cells and the unimpressive *in vitro* growth rates that most isolated angiosarcoma cell lines exhibit, we sought to evaluate optimal ECM substrate preference of these tumor cells to enhance their growth in culture

## RESULTS

**Figure 1:**
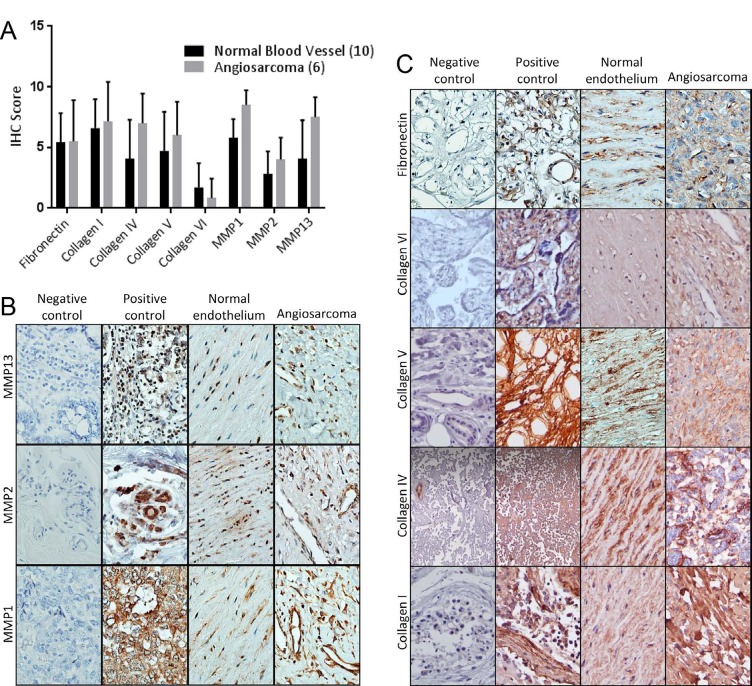
Expression of extracellular matrix components and their regulators in angiosarcoma and non-diseased endothelial tissues Angiosarcoma (N=6) and non-diseased endothelial tissues (N=10) were subjected to IHC for detection of the levels of extracellular matrix proteins and their regulators. (A) IHC scores for the detected antigens. For statistical analysis, the Mann-Whitney rank sum test was used. Statistical significance was determined if the two-sided p value of the test was < 0.05. (B & C) Representative images of IHC antigenicity for MMPs (B) and extracellular matrix components (C) known to be expressed in cells of endothelial origin. Red/brown staining depicts positive antigenicity.

We compared the expression of angiosarcoma ECM proteins and their regulators to those found in non- diseased endothelium, observing positive antigenicity for fibronectin, collagen I, collagen IV, collagen V, collagen VI, MMP1, MMP2, and MMP13 in 6 angiosarcoma tumors and 10 non-diseased vascular tissues (Figure 1A). Wide variability in protein staining was observed for both normal and diseased endothelial cells, and statistical analysis of the quantitative IHC data revealed no significant difference in the expression of ECM proteins and their regulators between these tissues. Representative images of MMPs and ECM components are seen in Figures 1B and C, respectively.

**Figure 2:**
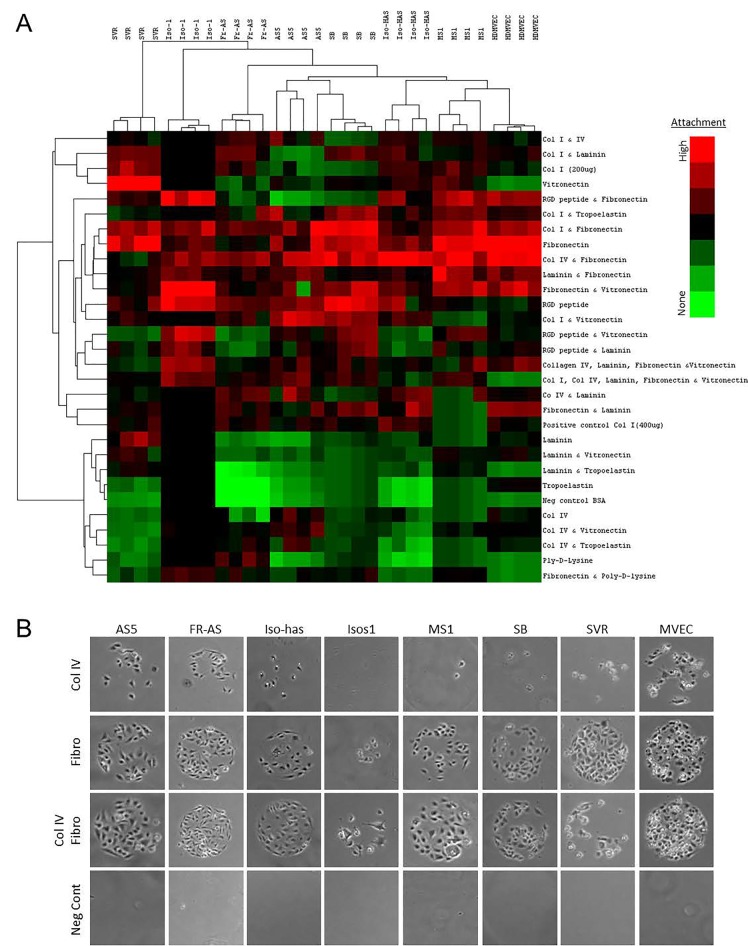
Extracellular matrix attachment preference of angiosarcoma cells Six angiosarcoma and 2 non-diseased endothelial cell lines were plated on extracellular matrix compositions deposited in quadruplicate onto a hydrogel surface as printed array spots. Adhesion was quantified at 30 minutes, whereby cell number was quantified on each array spot. (A) Heatmap depicting cell attachment to the extracellular matrix compositions. (B) Representative images of each cell lines adhering to highly preferred substrates (fibronectin) or less preferred substrates (collagen IV).

To determine if angiosarcomas exhibit a preference for certain ECM components, we utilized an ECM screening array containing 30 ECM components/mixtures deposited onto a hydrogel surface as printed array spots. Angiosarcoma cell lines tested included SVR (Ras- transformed mouse pancreatic endothelial cell line that forms aggressive angiosarcoma tumors in mice), Isos1 (murine-phenotypic angisarcoma cell line), FR-AS (canine hemangiosarcoma cell line), SB (canine hemangiosarcoma cell line), Iso-has (human scalp angiosarcoma cell line), and AS5 (human thigh angiosarcoma cell line). As controls we included a non-diseased primary human dermal microvascular endothelial cell line (HDMVEC) and a SV40 immortalized mouse pancreatic endothelium cell line (MS1). Both the angiosarcoma and non-diseased endothelial cells exhibited surprisingly similar attachment preferences for ECM substrates, with strong preference for collagen I and fibronectin, and less preference for collagen IV, laminin, and tropoelastin (Figure 2A). Representative images of each cell line on collagen IV, fibronectin, or the combination of both ECM components is provided in Figure 2B.

**Figure 3:**
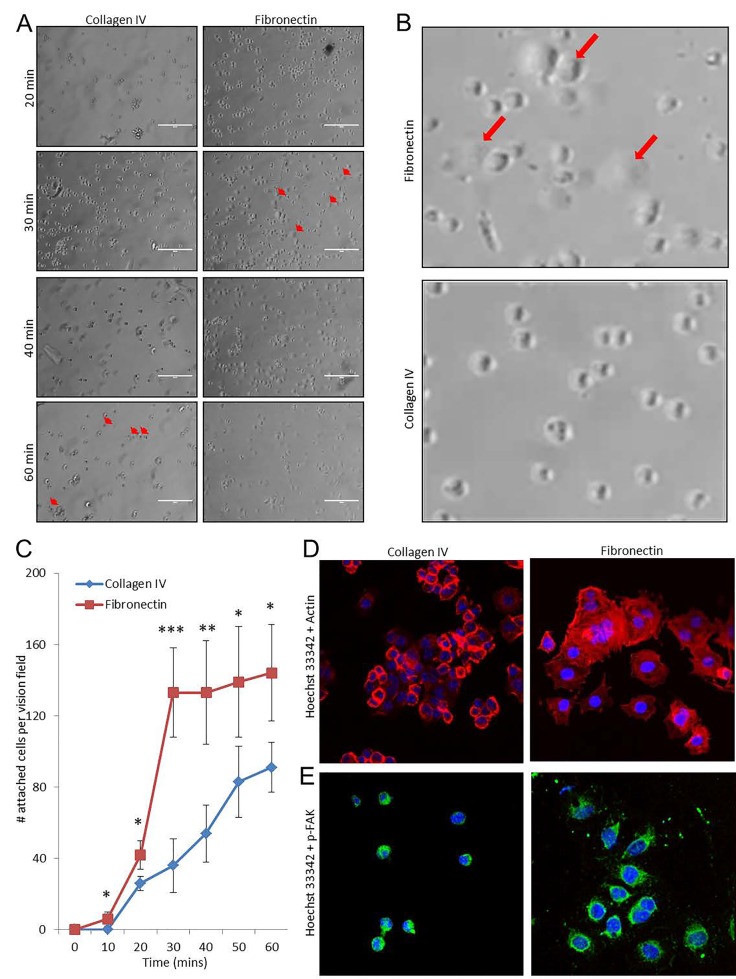
Timing of angiosarcoma cell adhesion to extracellular matrix substrates SVR cells were plated on 24 well plates coated with highly preferred substrates (fibronectin) or less preferred substrates (collagen IV), and images were taken over a course of 60 minutes (A, 200x magnification; B, 1000x magnification). (C) Cell attachment as a function of time was quantified for each substrate based on cell counts. Statistical analysis was performed using the Student's t-test method. Statistical significance was determined if the two-sided p value of the test was < 0.05. Immunofluorescent imaging of actin stress fibers (D) and phospho-focal adhesion kinase (E) in SVR cells at 60 minutes post-seeding on fibronectin or collagen IV substrates. Hoechst 33342 dye was used as a nuclear counterstain.

To evaluate the kinetics of angiosarcoma cell attachment to fibronectin (a preferred attachment substrate) and collagen IV (a less preferred attachment substrate), SVR cells were plated on wells pre-coated with either fibronectin or collagen IV, and images were taken of the cells every 10 minutes for one hour (Figure 3A-C). At 30 minutes, attachment of SVR cells to fibronectin began to plateau, suggesting that most of these cells had already adhered to the substrate by this time. In contrast, cell attachment to collagen IV had not yet plateaued by 60 minutes, suggesting that attachment significantly lagged behind those adhering to fibronectin. Similar results were observed for a panel of normal endothelial and angiosarcoma cells (Supplemental Figure 1). Attachment and spreading of SVR cells on fibronectin and collagen IV substrates was corroborated via immunofluorescence analysis of actin stress fibers (Figure 3D) and p-FAK (Figure 3E) at 60 minutes post cell seeding, revealing that cell spreading was increased when cells were plated on fibronectin.

**Figure 4:**
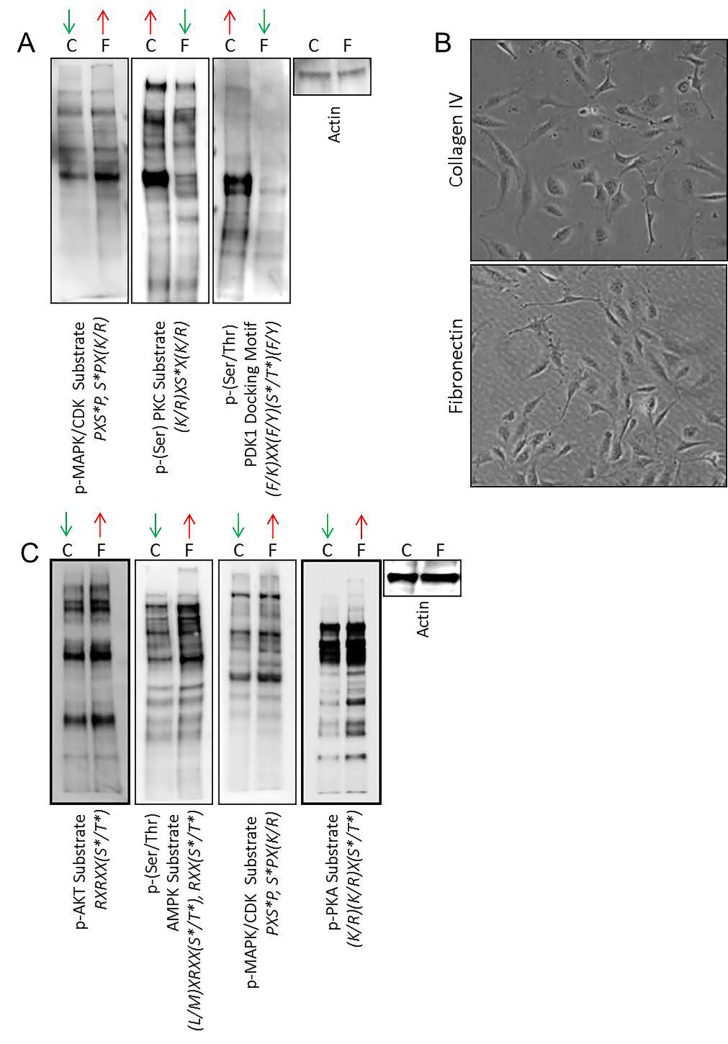
Extracellular matrix-mediated changes in intracellular signaling (A) SVR cells were plated on highly preferred substrates (fibronectin) or less preferred substrates (collagen IV) for 60 minutes. Cell lysates were collected and subjected to immunoblotting with phospho-motif antibodies against MAPK/CDK, PKC, or PDK1 downstream substrates. Beta-actin was used as a normalization control. Up/down arrows indicate if substrates of the evaluated signaling pathway show increased/decreased phosphorylation relative to the opposing treatment. (B) Images at 400x magnification of SVR cell attachment to fibronectin or collagen IV at 24 hours post-seeding. (C) SVR cells were plated on highly preferred substrates (fibronectin) or less preferred substrates (collagen IV) for 24 hours. Cell lysates were collected and subjected to immunoblotting with phospho-motif antibodies against AKT, AMPK, MAPK/CDK, or PKA downstream substrates. Beta-actin was used as a normalization control. Up/down arrows indicate if substrates of the evaluated signaling pathway show increased/decreased phosphorylation relative to the opposing treatment.

To evaluate the short-term intracellular signaling responses of angiosarcoma cells when plated on fibronectin verses collagen IV, we collected protein lysates from SVR cells at 60 minutes post-seeding on each substrate, and performed immunoblots using a set of phospho-motif antibodies that cover a large portion of the kinome regulated by diverse kinase families. Cells plated on fibronectin were characterized by increased phosphorylation of MAPK substrates and decreased phosphorylation of PKC and PDK1 substrates (Figure 4A). No change in phosphorylation patterns were observed for other kinome families (Supplemental Figure 2A).

We sought to determine if the acute adhesion properties observed in our experiments translated into longer term phenotypic changes that could enhance the *in vitro* growth properties of angiosarcoma cells. SVR cells exhibited equivalent adhesion on both fibronectin and collagen IV substrates at 24 hours post-seeding (Figure 4B), yet at this time point the cells growing on fibronectin exhibited increased AKT, AMPK, MAPK, and PKA substrate phosphorylation (Figure 4C). No change in phosphorylation patterns were seen for other kinome families (Supplemental Figure 2B).

**Figure 5:**
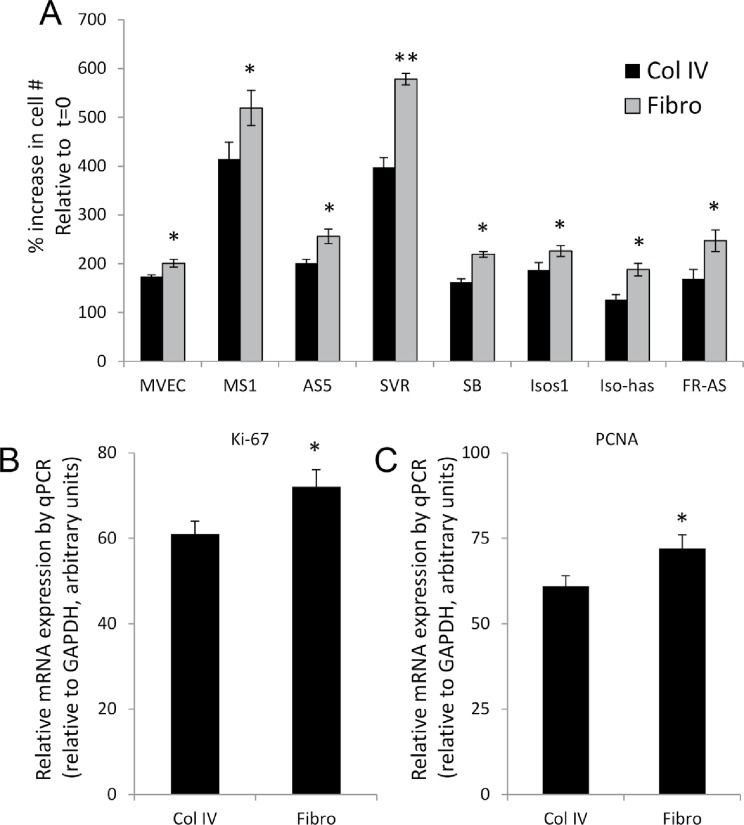
Proliferation rates of angiosarcoma cells are dependent on the extracellular matrix. Six angiosarcoma and two non-diseased endothelial cell lines were plated on highly preferred substrates (fibronectin) or less preferred substrates (collagen IV) for 48 hours. (A) Cell number was quantified using manual counts. All data are depicted as the % change in cell number at 48 hours relative to the time of initial plating. (B & C) qPCR detection of Ki-67 (B) and PCNA (C) mRNA in SVR cells after 48 hours growth on fibronectin or collagen IV. Statistical analysis was performed using the Student's t-test method. Statistical significance was determined if the two-sided p value of the test was < 0.05.

Given the influence of ECM composition on sustained intracellular signaling changes in angiosarcoma cells, we evaluated the effect of fibronectin verses collagen IV on cell proliferation using our panel of angiosarcoma cell lines and non-diseased endothelial cell controls. Though each cell line displayed markedly different overall growth rates, in every case increased proliferative rates were observed when cells were grown on fibronectin as opposed to collagen IV (Figure 5A). We corroborated this finding by evaluating the mRNA expression of the proliferation markers Ki-67 and PCNA in SVR cells plated on fibronectin or collagen IV, revealing significant fibronectin-induced increases in both markers after 48 hours (Figures 5B & C).

## DISCUSSION

In this study, we evaluated ECM preferences in angiosarcoma cell lines, revealing distinct partiality for certain substrate components over others. These findings offer valuable information on ECM preference of vascular tumor cells and provide insightful methodology to guide future researchers in the generation and use of tumor cell lines from these very lethal and rare sarcomas.

Our lab has previously shown that aberrant endothelial cells isolated from the benign vascular tumor, infantile hemangioma, differ in their expression patterns and regulation of ECM components from that of normal endothelial cells [17, 18], though it is unclear if similar properties are present in malignant vascular tumors such as angiosarcomas. We demonstrate that multiple ECM proteins and their MMP regulators are detectable in angiosarcoma tissues. ECM components of the endothelial basement membrane as well as their MMP regulators have been previously reported in angiosarcoma, however immunoreactivity of these molecules was widely variable both in composition and structure in the tumor samples relative to normal endothelium [19-25]. Our findings corroborated these previous reports, showing that due to the high variability of protein immunoreactivity between tissues, no statistical difference was observed in their expression levels between normal and tumorigenic endothelium.

Angiosarcomas as well as non-diseased dermal microvascular endothelial cells exhibited highly similar extracellular matrix adhesion preferences based on our findings. These data, coupled with our IHC data, suggest that the structural microenvironment that supports angiosarcoma cell growth is likely not markedly different from that of normal endothelial cells, and growth conditions that typically support cultured endothelial cells may be optimal for the maintenance of isolated angiosarcoma cells. Our data suggests that less preferred substrates are not inhibitory to attachment in mixed compositions, as mixtures of less preferred and highly preferred adhesion substrates still result in strong angiosarcoma cell attachment. Should researchers in the future attempt to isolate and culture angiosarcoma cells from solid tumors, our data suggest these cells should be plated onto ECMs such as collagen I or fibronectin, while ECM components such as collagen IV, laminin, or tropoelastin should be avoided.

At the molecular level, we demonstrated that within one hour of attachment to fibronectin the angiosarcoma cells exhibited enhanced phosphorylation of MAPK/CDK substrates, and reduction in the phosphorylation of PKC and PDK1 substrates. Fibronectin has previously been shown to potentiate MAPK signaling in endothelial cells [26], suggesting that this ECM component promotes mitogenic signaling. Moreover, reductions in PDK1 and PKC on preferred adhesion substrates is logical, given that PDK1, an upstream regulator of PKC [27], promotes focal adhesion disassembly to enhance cell movement and invasion [28, 29]. Despite the short term attachment preference of angiosarcoma cells to fibronectin, the cells eventually adhered to collagen IV within a 24 hour period, yet mitogenic and survival signaling such AKT, AMPK, and MAPK/CDK pathways remained substantially enhanced on fibronectin compared to collagen IV substrates. Indeed, all angiosarcoma and non-diseased endothelial cells lines tested exhibited increased proliferation rates on fibronectin substrates compared to collagen IV. The responses of angiosarcoma cells to fibronectin are indicative of pro-survival, highly metabolic, mitogenic behavior, suggesting that the matrix composition of angiosarcoma cells contributes not only to substrate adhesion, but also influences angiosarcoma cell behavior in a sustained manner.

## MATERIALS AND METHODS

### Immunohistochemistry (IHC)

IHC was performed on 5 µm thick, formalin fixed, paraffin-embedded sections commercially obtained tumor tissue arrays (US Biomax, Inc.; #SO8010) consisting of 6 cases of angiosarcoma and 10 normal (aortic or carotid artery) blood vessel tissues. The pathological features of each tumor were confirmed independently by a University Medical Center Pathologist. Sections were deparaffinized, rehydrated, and treated for antigen retrieval using Trilogy (Cell Marque). Non-specific binding was blocked with background block solution (Cell Marque). Antigens were detected with antibodies purchased from Abcam as follows: anti-fibronectin (Abcam #ab2413), anti-collagen I (Abcam #ab34710), anti-collagen IV (Abcam #ab6586), anti-collagen V (Abcam (#ab7046), anti-collagen VI (Abcam #ab180855), anti-MMP1 (Abcam #ab38929), anti-MMP2 (Abcam #ab110186), anti-MMP13 (Abcam #ab39012). Sections were then incubated with the CytoScan Alkaline Phos Detection System (Cell Marque) and detected using the DAB substrate kit (Cell Marque). All slides were counterstained with Hematoxylin. Immunopositivity was quantified blindly using two metrics: the percentage of tissue with positive staining and the staining intensity on a scale of 0-3 for each attribute. IHC scores were determined by multiplying the staining intensity by the percent of tissue stained. For statistical analysis, the Mann-Whitney rank sum test was used. Statistical significance was determined if the two-sided p value of the test was < 0.05.

### Cell culture

MS1 (ATCC #CRL-2279) and SVR cells (ATCC #CRL-2280) were maintained in Dulbecco's modified Eagle's media (DMEM) supplemented with 10% fetal bovine serum (FBS), 80 U/ml penicillin, and 50 µg/ml streptomycin C. Non-diseased human dermal microvascular endothelial cells (HDMVECs; Lonza #CC- 2543), FR-AS (generous gift from J. Modiano, University of Minnesota), SB (generous gift from J. Modiano, University of Minnesota), Iso-has (generous gift from M. Masuzawa, Kitasato University School of Medicine), Isos1 (generous gift from M. Masuzawa, Kitasato University School of Medicine), and AS5 (generous gift from E. Dickerson, University of Minnesota) were maintained in endothelial cell growth medium supplemented with the EGM-2 BulletKit (Lonza #CC-3162).

### Extracellular matrix adhesion arrays

Non-diseased and malignant endothelial cells were trypsinized, suspended in their respective growth media, and 150,000 cells were seeded onto the ECM Select Array Kit (Advanced Biomatrix #5171) which contained thirty extracellular matrix compositions deposited in quadruplicate onto a hydrogel surface as printed array spots (Kuschel Biotechniques 2006). The arrays were washed 2x at 30 minutes post-seeding in Hank's Balanced Salt Solution (ThermoFisher #14025092) and fixed in 4% paraformaldehyde. Images of each array spot were captured using a digital microscope and cells were counted for each spot. Data was input into a tab- delimited Microscope Excel file and imported into Cluster 3.0 software (http://bonsai.hgc.jp/∼mdehoon/software/cluster/software.htm) where data was centered, normalized, and centroid linkage clustering was performed with a correlation similarity metric. *.CDT files generated in Cluster 3.0 were imported into Java TreeView (http://jtreeview.sourceforge.net/) and heatmaps were generated to represent the quadruplicate attachment preferences of the cell lines for the extracellular matrix compositions on the ECM array.

### Quantifying SVR attachment to collagen IV and fibronectin

Cells were trypsinized, suspended in their respective growth media, and 150,000 cells were seeded onto collagen IV or fibronectin coated tissue (6) well culture dishes. Images of attached cells were taken with a digital microscope every 10 minutes for the indicated time points. Statistical analysis of attachment was performed using the Student's t-test method. Statistical significance was determined if the two-sided p value of the test was < 0.05.

### Immunofluorescent staining

Cells were plated for 60 minutes on collagen IV- or fibronectin-coated glass coverslips and fixed for 10 minutes in 4% paraformaldehyde solution. The cells were permeabilized with 0.1% Triton X-100 and blocked in 1% BSA. For actin visualization, coverslips were incubated for 20 minutes with fluorescent rhodamine phallotoxins (Life Technologies #R415). Phospho-FAK was detected with a primary antibody (Cell Signaling Technology #3283) and fluorescently labeled with a secondary anti- rabbit antibody. As a nuclear counterstain, cells were incubated with Hoechst 33342 dye. Fluorescent images were captured on a Nikon confocal microscope.

### Immunoblots using KinomeView Profiling Kit

Cells were plated for 60 minutes on collagen IV- or fibronectin-coated flasks. Cell lysates were then collected and immunoblots were performed using the Phospho- motif primary antibodies provided in the KinomeView Kit (Cell Signaling Technology #9812). Appropriate secondary antibodies were used to detect the signaling motifs on each immunoblot. Final visualization was achieved via incubation with Supersignal West Dura kit (Thermo Scientific) and images were taken with Fuji digital film imaging station.

### Cell proliferation/survival

Cells were plated at approximately 25% confluence on collagen IV or fibronectin coated (24) well plates. Time lapse microscopy was performed using a BioStation CT (Nikon) and the change in cell number after 48 hours was determined by manually counting cells per vision field. Numerical data presented is the average of at least three biological replicates +/- the standard deviation. Statistical analysis of attachment was performed using the Student's t-test method. Statistical significance was determined if the two-sided p value of the test was < 0.05.

### Quantitative PCR analysis (qPCR)

Cells were grown in triplicate biological replicates for 48 hours on collagen IV or fibronectin coated flasks. Total RNA was isolated using the Purelink RNA Micro kit (Invitrogen). RNA was then converted to cDNA using the Verso cDNA kit (Thermo-Scientific) and qPCR was performed using SYBR Green probes (Invitrogen) with an ABI7900HT real time PCR instrument (Invitrogen).

## SUPPLEMENTARY MATERIALS FIGURES



## Abbreviations

AMPK: 5′ adenosine monophosphate-activated protein kinase
ATM: Ataxia telangiectasia mutated
BSA: Bovine serum albumin
CDK: Cyclin dependent kinase
cDNA: complementary deoxyribonucleic acid
CK2: Casein kinase 2
DMEM: Dulbecco's Modified Eagle's Medium
ECM: Extracellular matrix
HDMVEC: Human dermal microvascular endothelial cells
IHC: Immunohistochemistry
Ki-67: Proliferation related Ki-67 antigen
MAPK: Mitogen activated protein kinase
MMP: Matrix metalloproteinase
mRNA: messenger ribonucleic acid
PCNA: Proliferating cell nuclear antigen
PCR: Polymerase chain reaction
PDK1: Phosphoinositide-dependent kinase 1
p-FAK: Phospho-focal adhesion kinase
PKA: Protein kinase A
PKC: Protein kinase C
PLC: Phospholipase C
qPCR: Quantitative polymerase chain reaction
RNA: Ribonucleic acid
SV40: Simian vacuolating virus 40
TNM: Tumor, node, metastasis
