# Supplementary material for: Extracellular matrix composition modulates angiosarcoma cell attachment and proliferation
Source: Oncoscience. 2017 Dec 7;4(11-12):178–88. doi: 10.18632/oncoscience.383 (PMC5769982; doi:10.18632/oncoscience.383)
Supplement: Supplementary file 1 [file oncoscience-04-0178-s001.pdf]

SUPPLEMENTARY MATERIALS

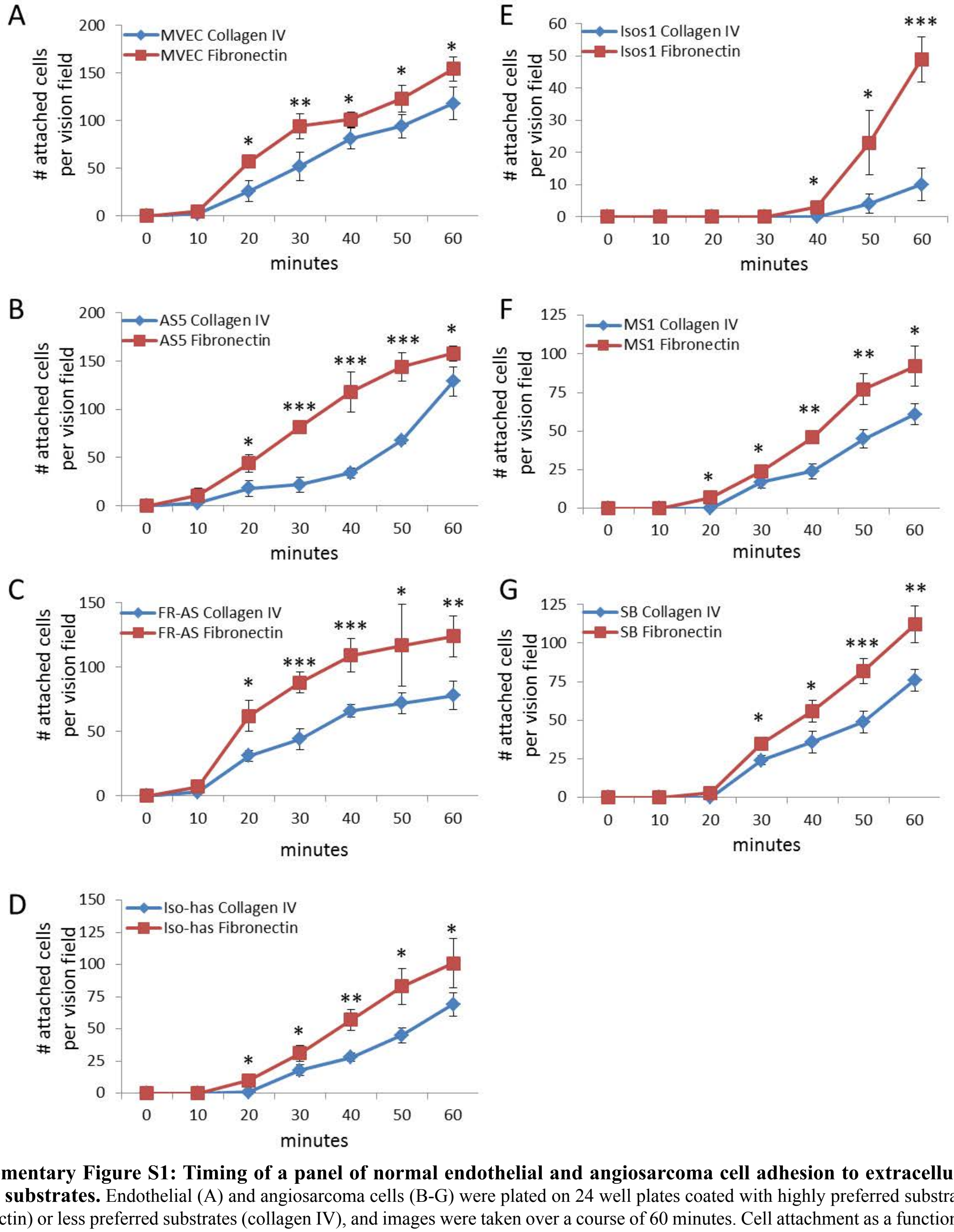

Supplementary Figure S1: Timing of a panel of normal endothelial and angiosarcoma cell adhesion to extracellular matrix substrates. Endothelial (A) and angiosarcoma cells (B-G) were plated on 24 well plates coated with highly preferred substrates (fibronectin) or less preferred substrates (collagen IV), and images were taken over a course of 60 minutes. Cell attachment as a function of time was quantified for each substrate based on cell counts. Statistical analysis was performed using the Student's t-test method. Statistical significance was determined if the two-sided p value of the test was < 0.05.

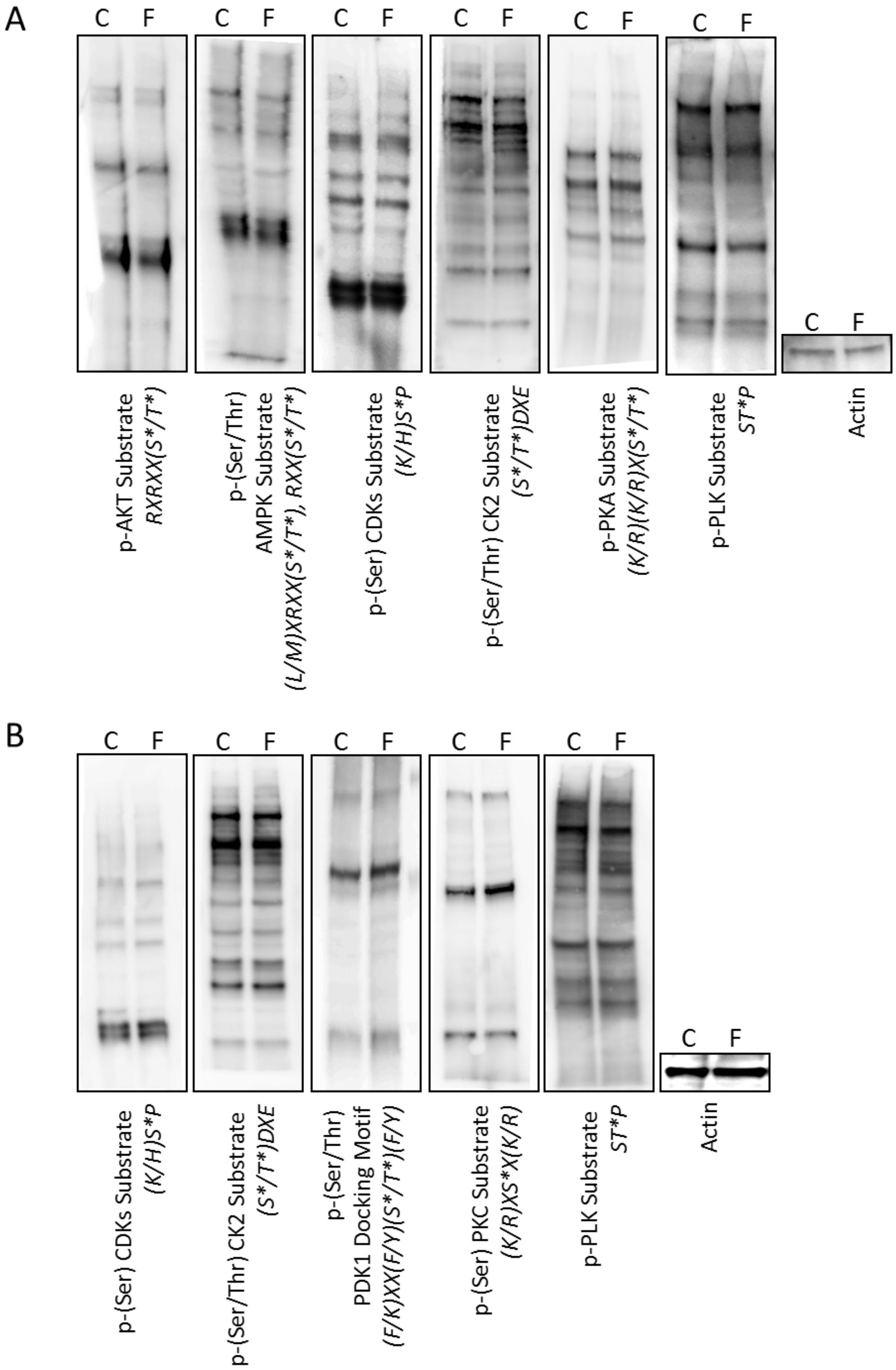

Supplementary Figure S2: Intracellular signaling cascades not affected by extracellular matrix. SVR cells were plated on highly preferred substrates (fibronectin) or less preferred substrates (collagen IV) for 60 minutes (A) or 24 hours (B). Cell lysates were collected at each time point and subjected to immunoblotting with phospho-motif antibodies. Beta-actin was used as a normalization control.
